# Anatomy and clinical relevance of sub occipital soft tissue connections with the dura mater in the upper cervical spine

**DOI:** 10.7717/peerj.9716

**Published:** 2020-08-10

**Authors:** Rob Sillevis, Russell Hogg

**Affiliations:** Department of Rehabilitation Sciences, Florida Gulf Coast University, Fort Myers, FL, USA

**Keywords:** Dissection, Myodural bridge, Nuchal ligament, Cervical spine

## Abstract

**Background:**

The upper cervical region is a complex anatomical structure. Myodural bridges between posterior suboccipital muscles and the dura might be important explaining conditions associated with the upper cervical spine dysfunction such as cervicogenic headache. This cadaver study explored the upper cervical spine and evaluated the myodural bridges along with position of spinal cord in response to passive motion of upper cervical spine.

**Methods:**

A total of seven adult cadavers were used in this exploratory study. The suboccipital muscles and nuchal ligament were exposed. Connections between the Rectus Capitis Posterior major/minor and the Obliquus Capitis minor, the nuchal ligament, posterior aspect of the cervical spine, flavum ligament and the dura were explored and confirmed with histology. The position of the spinal cord was evaluated with passive motions of the upper cervical spine.

**Outcomes:**

In all cadavers connective tissues attaching the Rectus Capitis Posterior Major to the posterior atlanto-occipital membrane were identified. In the sagittal dissection we observed connection between the nuchal ligament and the dura. Histology revealed that the connection is collagenous in nature. The spinal cord moves within the spinal canal during passive movement.

**Discussion:**

The presence of tissue connections between ligament, bone and muscles in the suboccipital region was confirmed. The nuchal ligament was continuous with the menigiovertebral ligament and the dura. Passive upper cervical motion results in spinal cord motion within the canal and possible tensioning of nerve and ligamentous connections.

## Introduction

The relationship between the upper cervical spine and headaches has been established (Castien, De Hertogh & Scholten-Peeters, 2016; Fernandez-de-Las-Penas & Cuadrado, 2016). Musculoskeletal dysfunction including neurological symptoms, such as muscle weakness and paresthesia in the occipital nerve distribution region, are common in headaches, especially in tension type and cervicogenic headaches (Caamano-Barrios et al., 2019). About 15–20% of all headaches are cervicogenic in nature and the prevalence in the general population is around 4%. Cervicogenic headaches appear to originate in the high cervical spine and typically result in unilateral pain in the head and face. The International Headache Society defines cervicogenic headache as “pain, referred from a structure in the neck” (Headache Classification Subcommittee of the International Headache Society, 2004). The exact mechanism by which cervicogenic headaches are caused is not clear. A possible rationale is the direct relationship between the spinal nerves of C1–C3 and the trigeminal nerve at the trigeminocervical nucleus (Castien, De Hertogh & Scholten-Peeters, 2016; Fernandez-de-Las-Penas & Cuadrado, 2016).

Any structure in the upper cervical spine such as the intervertebral disc, spinal ligaments, muscles, articulation, dura, and nerve roots could be a generating pain source and causing musculoskeletal dysfunction leading to cervicogenic headaches (Bogduk, 1994; Krauss et al., 2008). Movement of the cervical spine is dependent on the shape and spatial orientation of articular surfaces, the stabilizing ligaments, and muscles controlling the region. Intervertebral motion is quite similar in the mid and lower cervical spine but varies greatly in the upper cervical region (Occiput-C3) (Wu et al., 2010). The C1–C2 segment contributes more than 50% of the total rotation of the neck. The motion in the upper cervical spine is controlled by several suboccipital muscles and stabilizing ligaments making sure that the integrity of this region is maintained.

**Figure 1:**
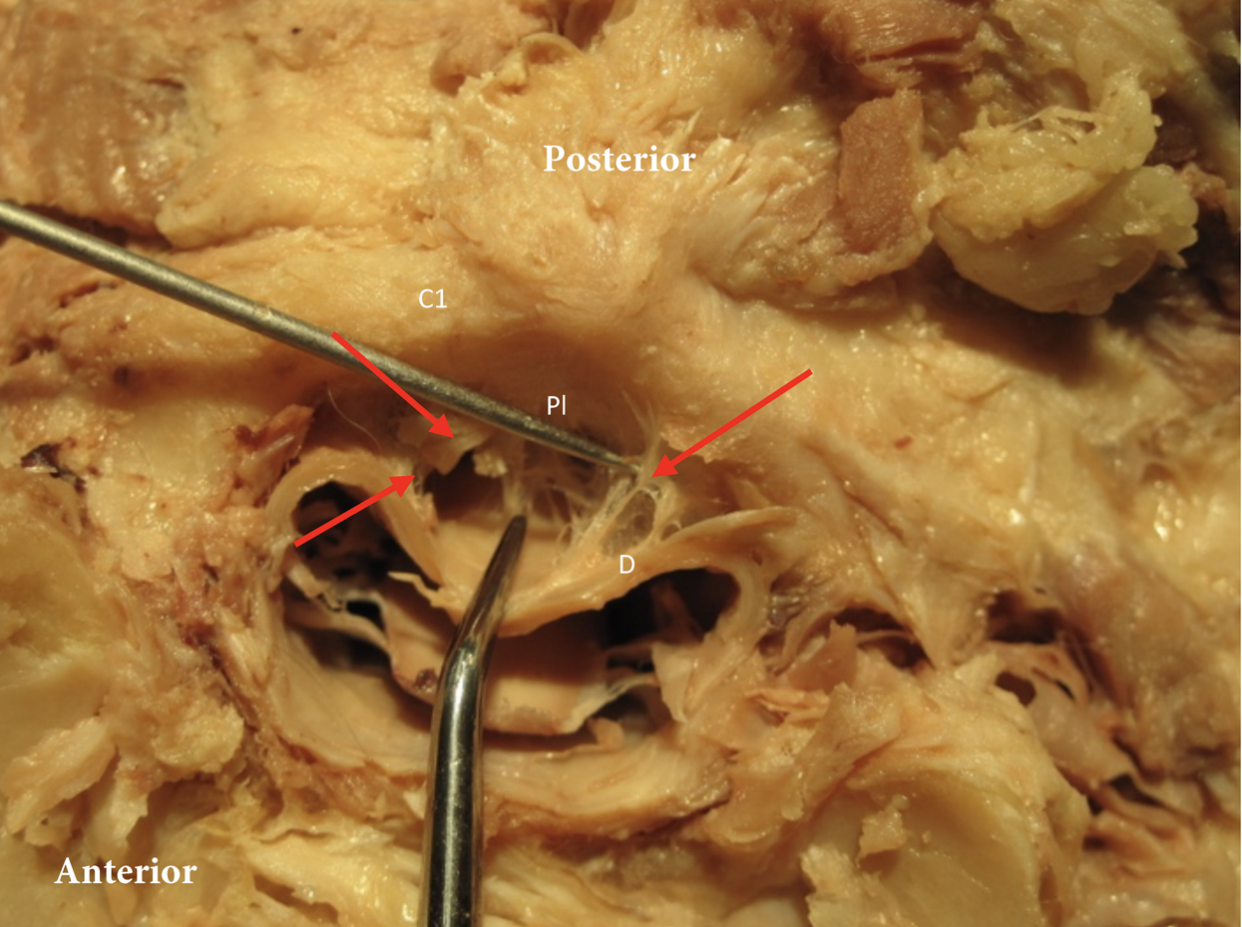
Posterior dura connection to posterior arch of C1. Posterior dura (D) connection to posterior arch of C1 (C1). Red arrows point to the connection between dura and the posterior longitudinal ligament (Pl).

In order to prevent compression of the dura mater during motion of the spinal column, the dura is anchored in the vertebral canal by multiple fibrous connections (Chen et al., 2015). These meningovertebral ligaments connect the anterior aspect of the dura to the posterior longitudinal ligament and the dorsal aspect of the dura to the ligamentum flavum or the lamina (Fig. 1) (Chen et al., 2015). It was demonstrated that the meningovertebral ligaments can be found all along the length of the vertebral column and that their shape varies greatly. They typically have one attachment on the dura and can be accompanied by vascular vessels. It was identified that these ligaments are composed of fibrous regular dense connective tissue (Chen et al., 2015).

**Figure 2:**
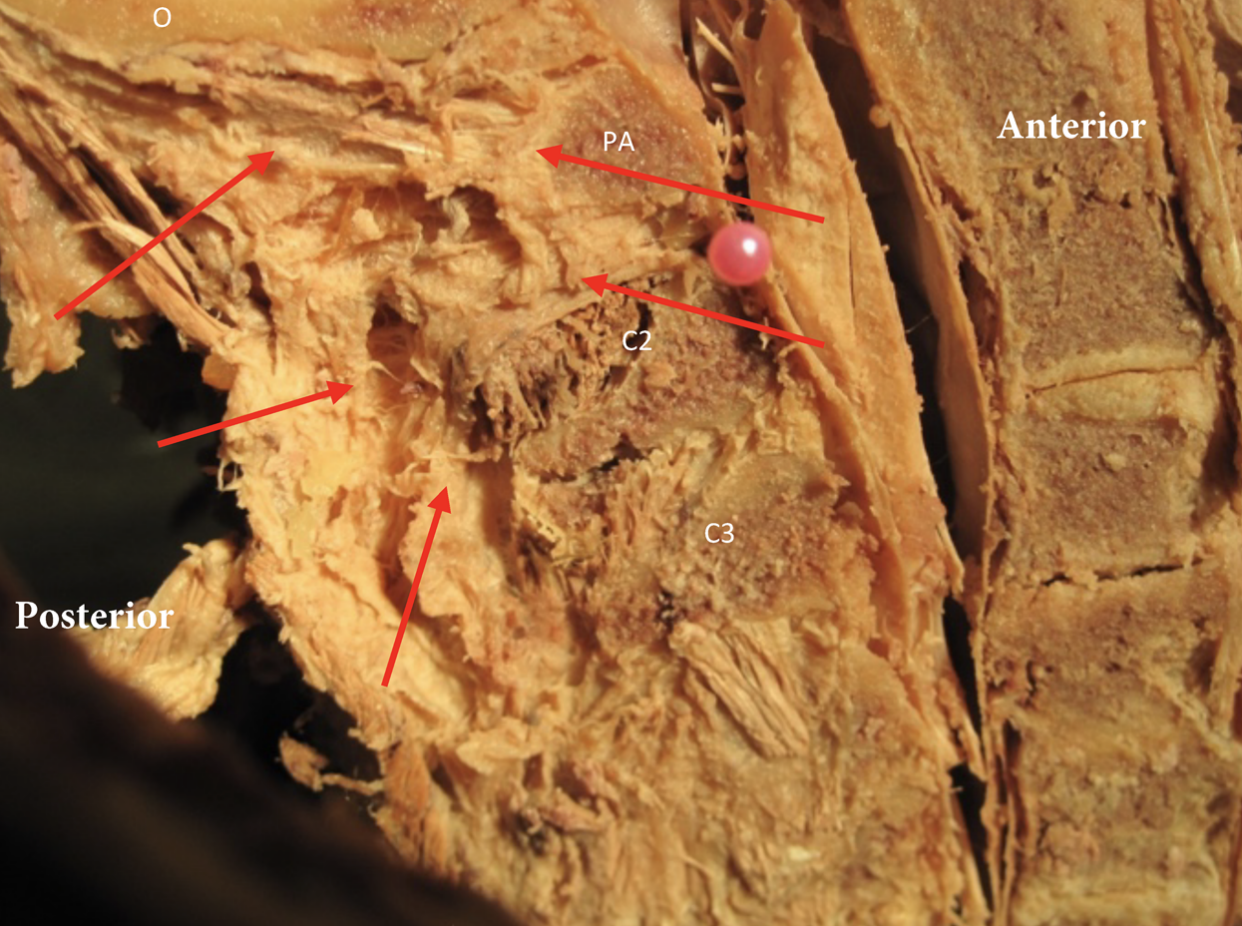
The nuchal ligament in the midsagittal plane and its orientation relative to the upper cervical spine. Red arrows indicate the ligament relative to the occipital bone (o), posterior arch of atlas (PA), spinous process C2 (C2) and C3 (C3).

Posterior to the spinal column is the nuchal ligament, the cephalic extension of the supraspinous ligament. It extends from the C7 spinous process and attaches to the cervical spinous processes and midsagittally along the occipital bone, terminating at the external occipital protuberance (Fig. 2). The majority of the nuchal ligament is formed from bilateral fascia contributions of the rhomboid minor, serratus posterior superior, splenius capitis and trapezius and thus might play a role in cervicogenic headaches (Kadri & Al-Mefty, 2007). The occipital insertion of the nuchal ligament cannot be clearly distinguished from adjoining fascial/tissue insertions. The nuchal ligament will restrict flexion of the cervical spine and it has been identified to contribute to the proprioceptive control of the high cervical region (Kadri & Al-Mefty, 2007).

In the upper cervical region the presence of fascial connections between the Rectus Capitis Posterior Major, Rectus Capitis Posterior Minor, and the Obliquus Capitis Inferior and the dura have been demonstrated (Scali, Marsili & Pontell, 2011; Pontell et al., 2013). These connections have been referred to as the “myodural bridges” (Scali, Marsili & Pontell, 2011; Pontell et al., 2013). Between the arches of C1 and C2, the myodural bridges merge with the meningovertebral ligaments and cross the epidural space, inserting into the posterior aspect of the dura mater. It was previously proposed that these myodural bridges are meant to protect the cervical dura during motion and prevent compression of the cord (Scali, Marsili & Pontell, 2011). The significance of this finding is that increased muscle tone might directly affect positioning of the dura in the spinal canal and it could be hypothesized that this might play a role in the development of cervicogenic headaches. A posterior pull of the dura can be expected with increased suboccipital muscle tone. This position will create tensioning of the anterior meningovertebral ligaments and might have an impact on dura tension during upper cervical movement. This could potentially explain positive neural tension testing such as the slump test in subjects with cervicogenic and tension type headache (Caamano-Barrios et al., 2019; Szikszay, Luedtke & Von Harry, 2018; Von Piekartz, Schouten & Aufdemkampe, 2007). It was the aim of this cadaver study to further explore if there were direct soft tissue connections between the nuchal ligament and the dura mater in the upper cervical region and use histology to identify the type of soft tissue connection with the dura. Additionally, the position of the spinal cord in the vertebral foramen of C1 during passive induced motion of upper cervical spine was examined. A better understanding of these anatomical relationships and response to movement might assist clinicians in their clinical reasoning when managing patients with neck pain and/or headaches.

## Methods

**Figure 3:**
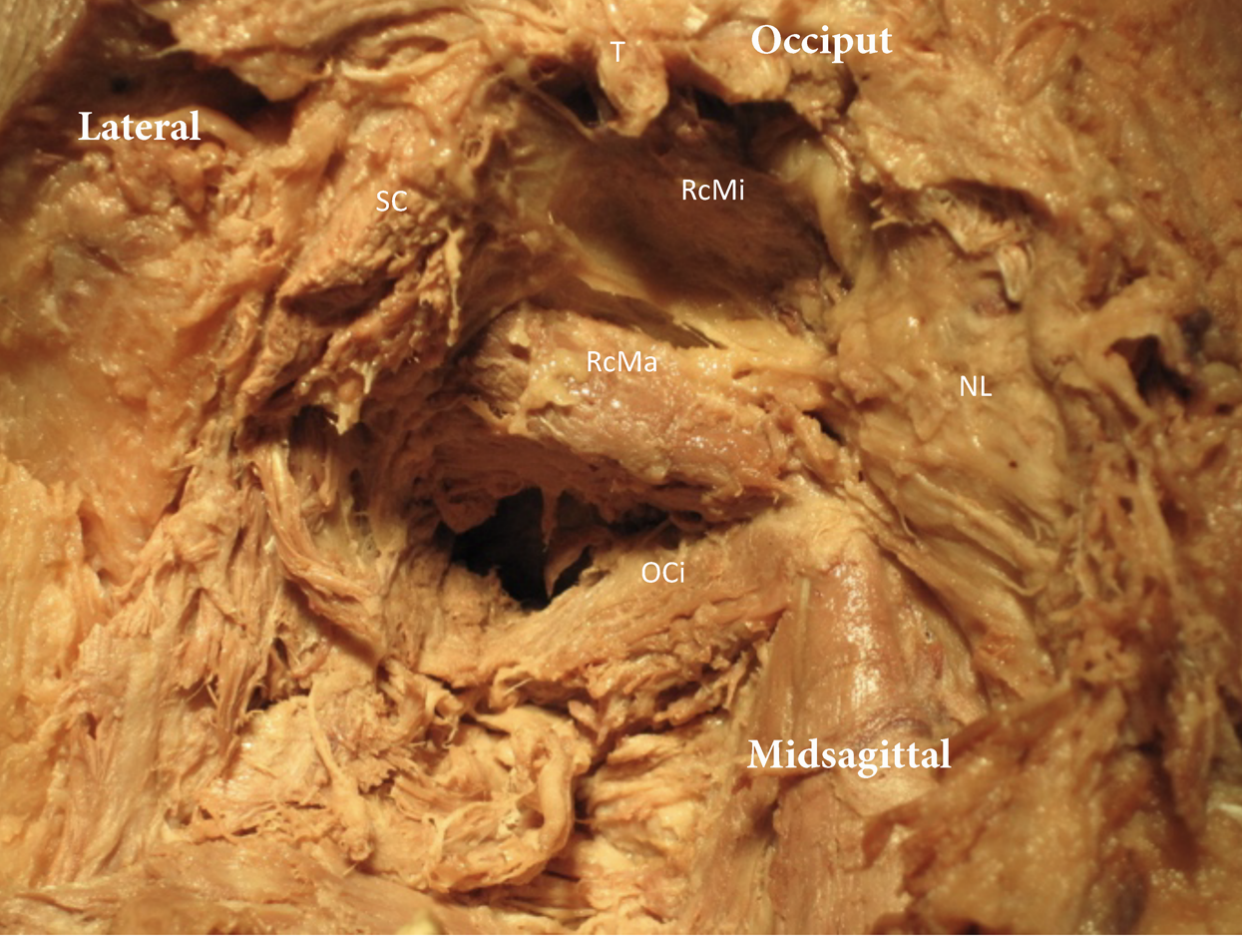
Exploration the suboccipital region. The trapezius (T) and Splenius Capitis (SC) have been removed. The Nuchal ligament (NL) is exposed and the Rectus Capitis Major (RcMa), Rectus Capitis Minor (RcMi) and Obliquus Capitis Inferior (OCi) can be seen.

A total of seven adult cadavers (three males, four females), all over the age of 50 were used in this exploratory study. All cadavers were acquired from the Anatomical Board of the State of Florida, following standard protocols according to Anatomical Board regulations and Florida State Law; all of them had been embalmed with a formalin-based solution. No medical history was known about any of the cadavers. Students of the Doctor of Physical Therapy program at Florida Gulf Coast University had previously performed dissections of the cadavers, though leaving the suboccipital region intact. The skin and superficial fascia over the posterior neck region was removed. Following this the trapezius and the splenius capitis were detached from the occiput, as was the semispinalis capitis. Following this the suboccipital muscles and the nuchal ligament were exposed (Fig. 3). The connections between the Rectus Capitis Posterior Major and Minor, the Obliquus Capitis Inferior and the nuchal ligament, posterior aspect of the cervical spine, flavum ligament and the dura were explored. To further confirm tissue connections between the posterior suboccipital muscles, nuchal ligament and the dura forceps were used. This allowed for a direct pull on muscle and ligamentous tissue. The intent of this action was to confirm the connection of the ligamentous tissue to the dura without quantifying the amount of force this connection could withstand. Therefore, the dissector determined the force used to explore this connection between muscle and dura and this varied from a gentle pull to a stronger pull.

After posterior exploration was completed, three specimens underwent a transverse cut between the occipital condyles and atlas, with an anterior reflection of the skull and pharynx, allowing for a direct transverse view of the spinal canal, dura and meningovertebral connections (Fig. 1). These three specimens were used to explore the effect of passive movement of the cervical spine and the position of the dura. In each specimen C1 was rotated right and left followed by flexion and extension of the cervical spine. At end range of passive motion, as allowed by the specimens, the spinal cord position relative to the central spinal canal was evaluated.

To determine what type of fibrous tissue connects the nuchal ligament to the dura we decided to further analyze this connection. In order to provide histological information, we cut the C1 and C2 segments, including all soft tissues, of one of these three specimens and decalcified it in an aqueous solution of sodium citrate and formic acid, until test samples of this solution added to an ammonium oxalate solution did not form any precipitate (the sample was checked weekly). Once decalcified, the specimen was embedded in a paraffin matrix and thin sectioned with a microtome to a thickness of five μm. Sections were mounted to glass slides and stained using Masson’s Trichrome staining protocol to identify collagen fibers in the final sections. Finally, specimens were imaged using transmitted light with a Zeiss Axioskop II microscope and Lumenera Infinity digital camera.

## Results

**Figure 4:**
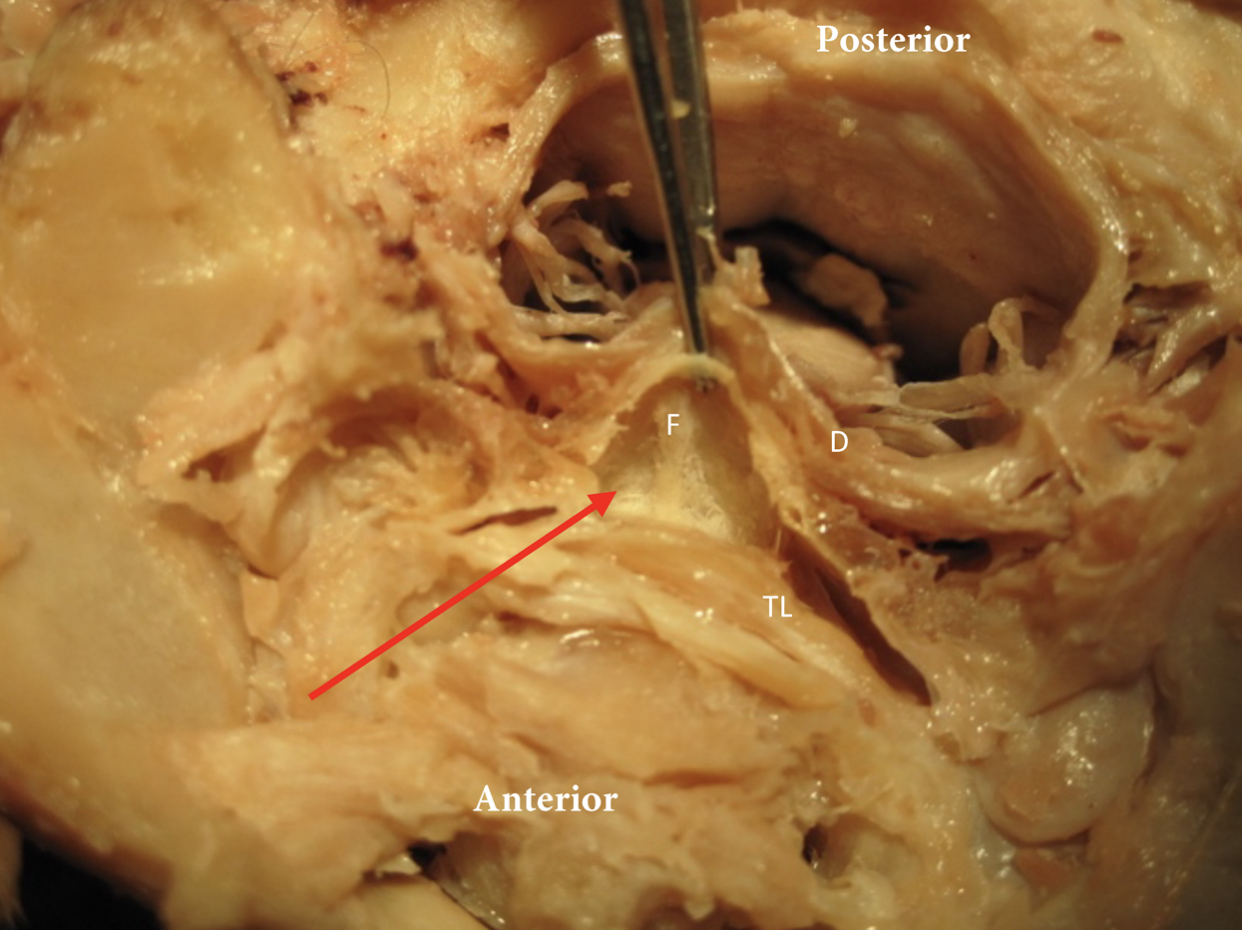
Meningovertebral fuzz. The red arrow identifies the tissue “Fuzz” (F) between posterior side transverse ligament (TL) and anterior aspect dura (D).

Our tissue samples as demonstrated in Figs. 1 and 2 indicate that there is a large network of tissues connections between ligaments, bone and muscles in the suboccipital region. It is clear that besides the meningovertebral ligament there are extensive loose areolar connective tissue connections (fuzz) between the dura and the posterior arch of atlas and the dura and the soft tissue between the dens and the dural sac (Fig. 4). It appeared consistently in our specimens that this fuzz could be fairly easily disrupted using the forceps and/or a probe.

**Figure 5:**
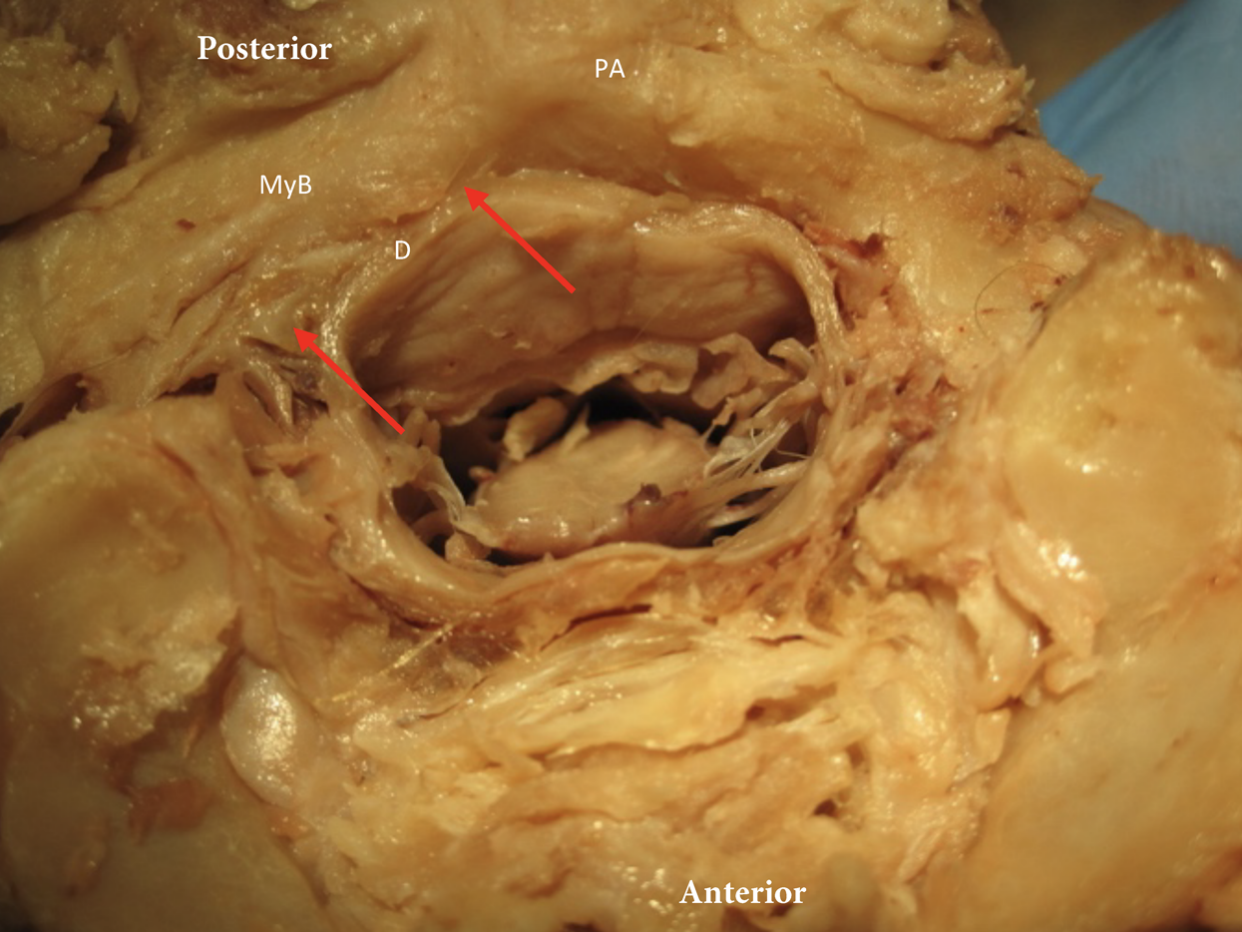
The myodural bridge. The myodural bridge (myB) is identified by the red arrows and spans over the posterior arch of C1 (PA) (right side identified) toward the dura (D).

The dissection of our specimens confirmed this that there is a deep fascial connection between the Rectus Capitis Major, Minor, and the Obliquus Capitis Inferior, and this appears to be blending with the dura in the spinal canal. The myodural bridge in our samples appeared fairly wide and covered a large portion of the posterior arch of atlas on each side (Fig. 5). In all cadavers it was possible to demonstrate connective tissue attaching the deep surface of the Rectus Capitis Posterior Major muscle to the transverse fibers of the posterior atlanto-occipital membrane, close to the margin of the foramen magnum. This connective tissue extended laterally and blended with the perivascular tissues of the vertebral arteries where they entered the cranium. There was no direct attachment observed of this connective tissue to the cervical spinal dura. However, when we provided tension to the rectus capitis muscles using forceps, we observed coincident movement in the dura mater.

**Figure 6:**
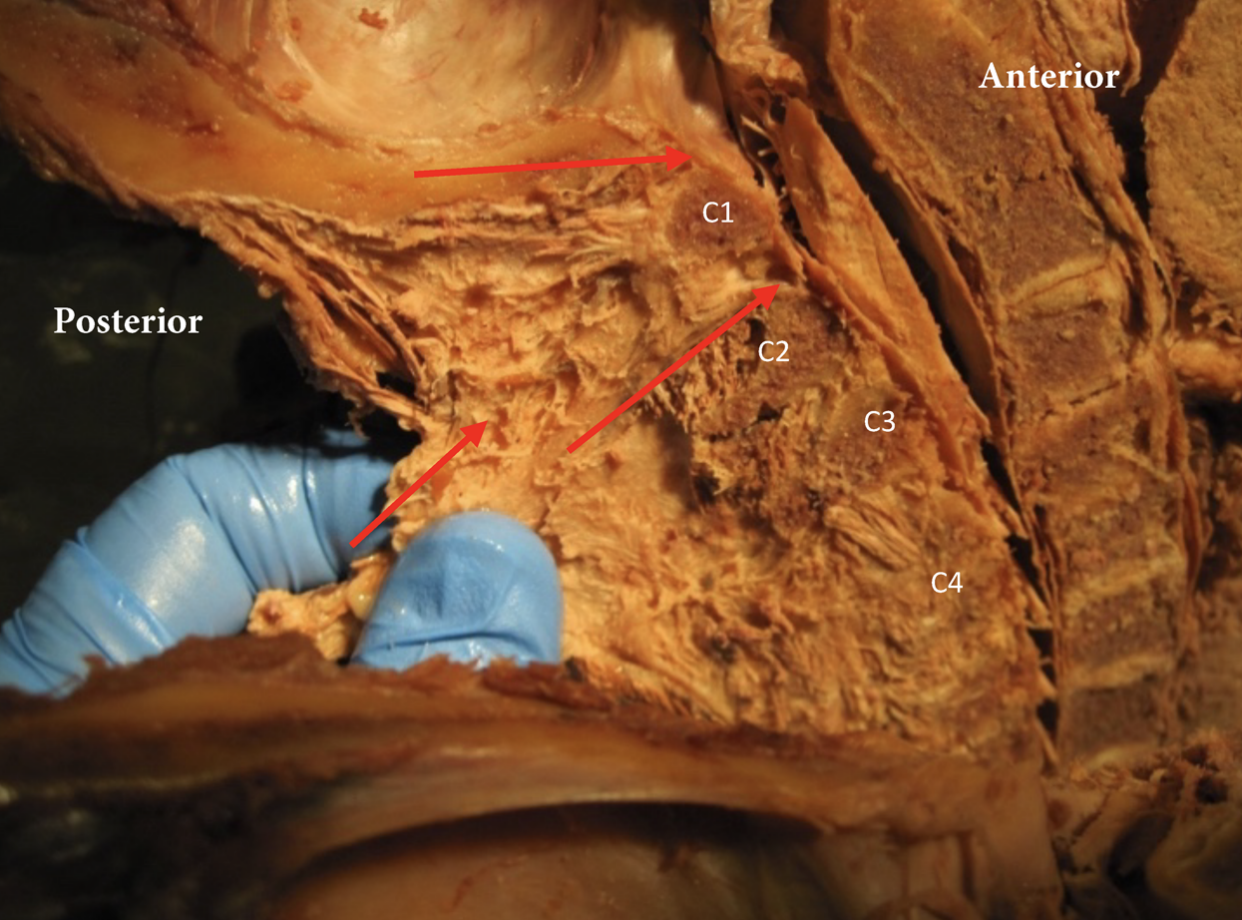
Nuchal ligament dural connection. Nuchal ligament (NL) with connection to atlas (A), spinous process C2 (C2), C3 (C3) and C4 (C4). Tensioning the nuchal line creates tension on dura (red arrows).

**Figure 7:**
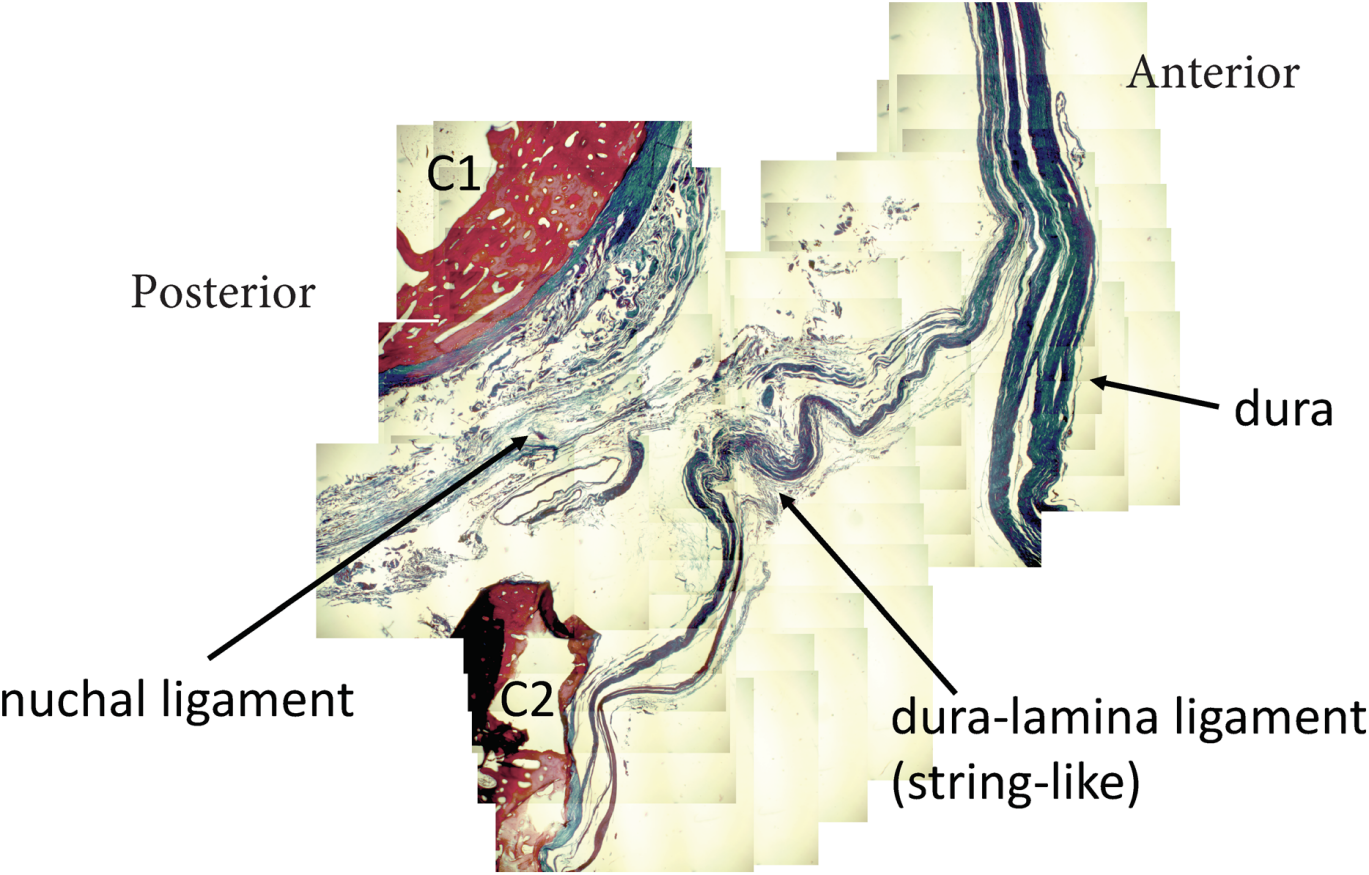
Nuchal-dural connection. Stained tissue slide demonstrating the presence of collagen fibers (blue) connecting the nuchal ligament with the meningovertebral ligament and blending with the dura.

In the sagittal dissection we observed that the nuchal ligament was continuous with the dura between the occipital bone and atlas and the atlas and axis (Figs. 6 and 7). A sample was taken from this connection (between C1 and C2) and the histology revealed that our sample was high in collagenous tissue similar to that of the nuchal ligament and the dura. Therefore, it was concluded that the nuchal ligament is part of a continuum with the same tissue consistency as the dura (Fig. 7). Additionally, a clear connective tissue strand with the same tissue formation was identified between the posterior arch of atlas and the dura. As seen in Fig. 7, the collagenous fibers of the meningovertrebral ligament blend with the collagenous fibers of the nuchal ligament tissue and become a continuous layer with the collagenous dura layer.

**Figure 8:**
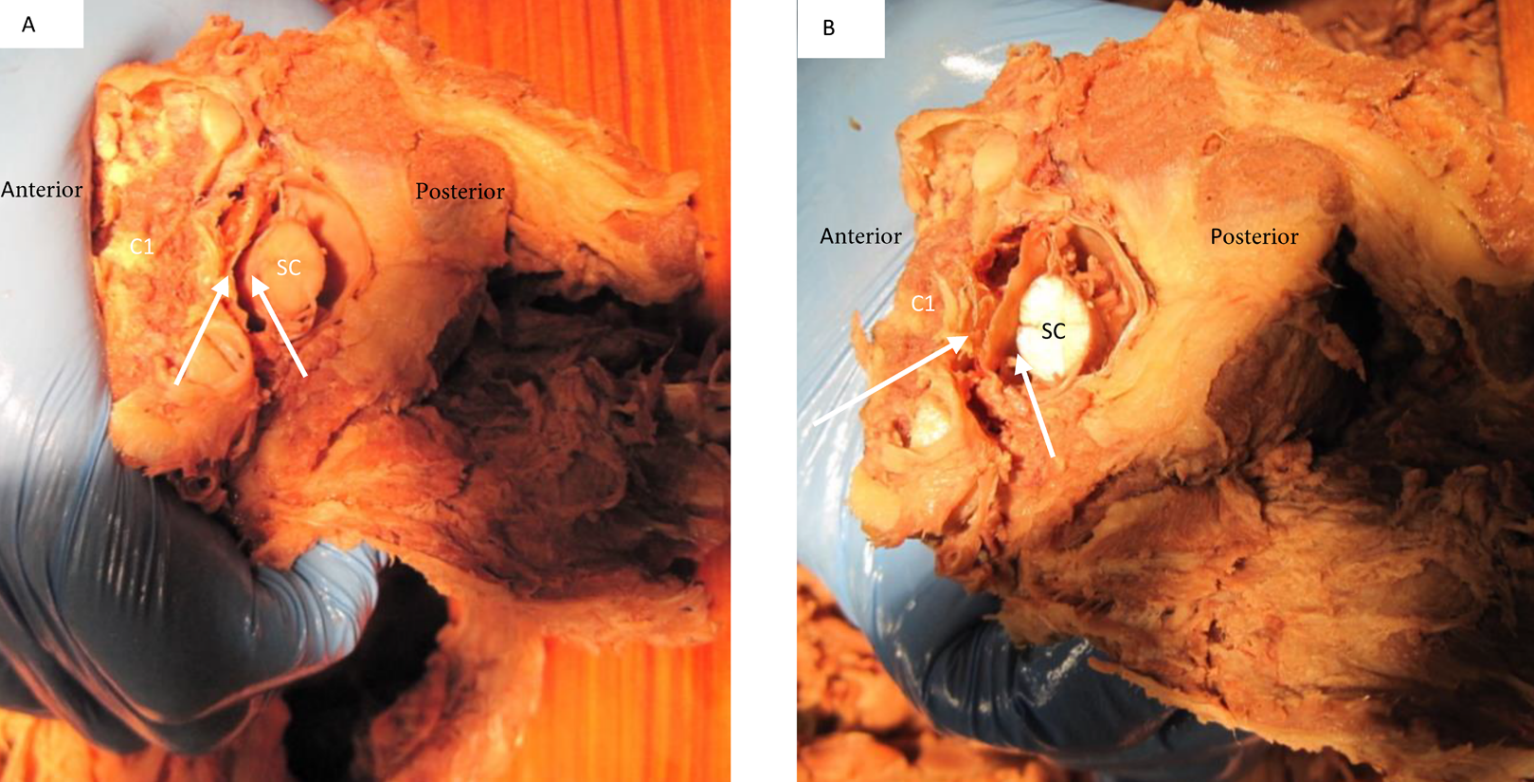
Deflection of spinal cord upon flexion and extension C1 on C2. In both images (A) signifies the anterior and P posterior aspect of the spine. Image (A) demonstrates the forward movement of the spinal cord (SC) during extension. The white arrows demonstrate the distance between anterior cord and posterior vertebral arch. Image (B) demonstrates a posterior deflection of the cord during flexion. The white arrows demonstrate the distance between anterior cord and posterior vertebral arch.

**Figure 9:**
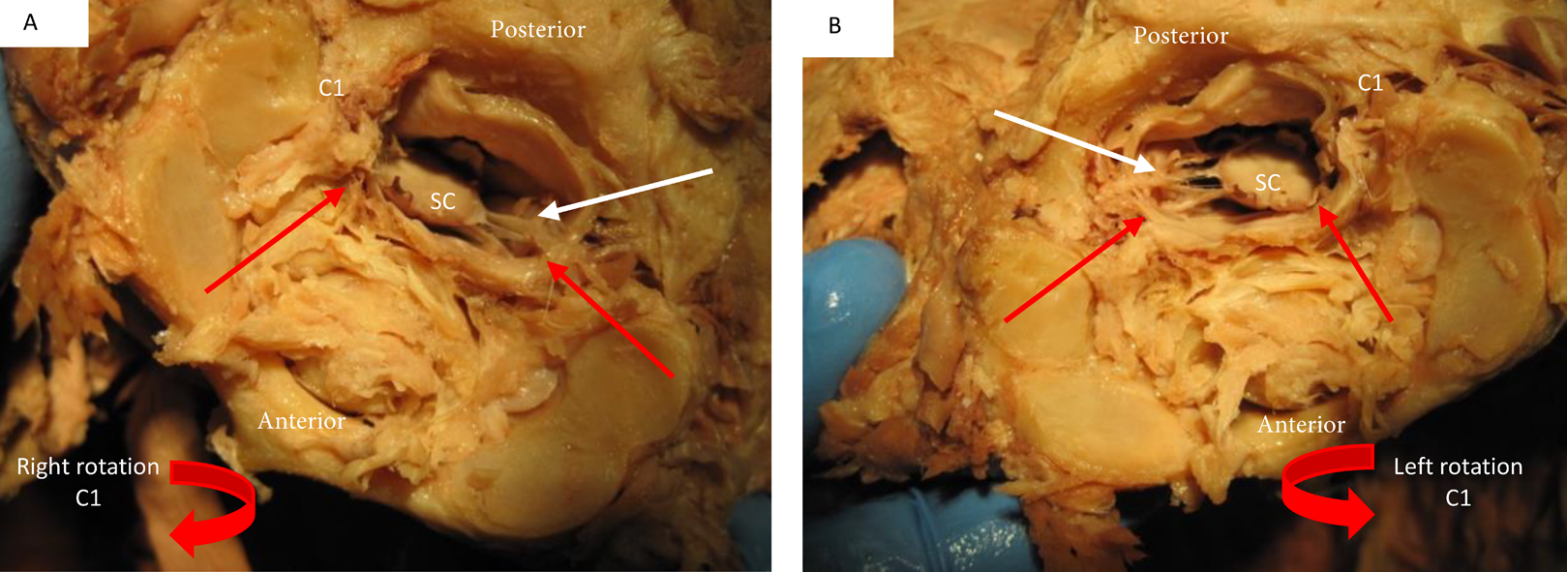
Deflection of spinal cord upon rotation C1 on C2. Image (A) demonstrates the movement of the spinal cord (SC) to the right during right rotation (red arrows demonstrate movement) relative to C1 (C1). The white arrow shows that the left spinal nerves appear tight during this motion. Image (B) demonstrates a left deflection of the cord during left rotation of C1 and there appears tightening of the right spinal nerves.

The dural sac and spinal cord moved within the spinal canal as a result of motion of the upper cervical spine. Flexion and extension resulted in forward and backward movement of the spinal cord respectively. Rotation resulted in homolateral movement and concurrent tensioning of the contralateral spinal nerve (Figs. 8 and 9).

## Discussion

The aim of this cadaver study was to explore the upper cervical region for soft tissue connections between the suboccipital musculature and ligaments and the dura mater. The superior cervical vertebral column is a very complex anatomical region. There appears to be a clear anatomical relationship between muscular, ligamentous, soft tissues and the dura mater in the high cervical region. Dissection both in the sagittal and the transverse plane demonstrates clearly that the epidural space of the upper cervical spine contains many connective tissues and fascia layers that merge and blend together regardless of their origination (Figs. 1 and 2). Our observations concur with the findings of Shi et al. (2014). They demonstrated that the intervertebral foramen contains intricate connections between the suboccipital musculature and the cervical dural sac. However, this is opposed to the traditional view that the atlantoaxial and atlanto-occipital membranes serve as a complete barrier between the suboccipital region and the contents of the vertebral canal (Scali et al., 2015).

It was observed that the nuchal ligament has a direct connection with the dura and blends with the meningovertebral ligament to blend with the dural sac. This observation concurs with previous reports about the nuchal ligament dural connection (Dean & Mitchell, 2002). Histology revealed that our tissue sample of the nuchal dural connection was high in collagenous tissue. It appeared similar to that of the nuchal ligament and the dura itself therefore supporting the notion this is a ligamentous connection (Fig. 7). The clinical consequence of this connection can only be speculated at this time. Neck flexion results in tensioning of the nuchal ligament and thus should also lead to a posterior pull of the dura. Our observations of the connections between the Rectus Capitis Posterior Major, Minor, and the Obliquus Capitis Inferior and the dura concur with previous reports (Pontell et al., 2013; Enix, Scali & Pontell, 2014).

The secondary aim of this study was to identify how the position of the spinal cord within the vertebral foramen of C1 changed during passively induced motion of upper cervical spine. When the cervical spine was extended in our cadavers it was observed that the spinal cord appeared to move forward within the spinal canal (Fig. 8). It can be hypothesized that this forward movement of the spinal cord would lead to the spinal nerves being pulled into the spinal canal to allow for this forward motion. During active movement of the cervical spine, the suboccipital musculature will control the motion between the occiput, C1 and C2. Neck extension of the upper cervical spine is the result of co-contraction of the bilateral Rectus Capitis Posterior Major and Minor, and the Obliquus Capitis Inferior (Klein et al., 2003). A suboccipital muscle contraction would result in a posterior pull on the dura through the myodural connections. This might prevent dural infoldings and would maintain a neutral cord position within the spinal canal without resulting in any pulling effect on the spinal nerves (Scali, Marsili & Pontell, 2011; Pontell et al., 2013). Passive flexion in our cadavers resulted in a backward motion of the spinal cord within the spinal canal. Both the “nuchal ligament” and the posterior meningovertebral ligaments could be responsible for this. The anterior meningovertebral ligaments between the anterior dura and the anterior arch of atlas might prevent excessive spinal cord motion. Additional research is necessary to determine the presence of myodural bridges between the anterior suboccipital muscles and the dura. The presence of such connections might assist in preventing excessive movement and possible infolding of the spinal cord position posteriorly.

When C1 was rotated on C2 in our specimens we observed that there was an ipsilateral movement of the spinal cord within the spinal canal (Fig. 9). As seen in this image this spinal cord movement resulted in visual tensioning of the contralateral spinal nerve at this level. Shi et al. (2014) reported the presence of transforaminal ligaments. It was proposed that they may serve to position the spinal nerve in the foraminal space optimally and protect it against a direct pulling force on the nerve due to movement of the arms and/or neck and head. Based on our observation the transforaminal ligament does not prevent motion of the spinal cord so this would support the thought of tensioning in the spinal nerve with rotation of the neck. The previous discussed myodural bridge between the suboccipital muscles might play an additional role during rotation of the neck. Rotation of C1 is the result of co-contraction of the homolateral Rectus Capitis Posterior Major, Minor and the Obliquus Capitis Inferior muscles. This would result in an additional pull on the spinal cord through the myodural bridge to the side of contraction. Our findings would concur with the findings of Scali, Marsili & Pontell (2011) and Pontell et al. (2013) who suggested that increased tension of the Rectus Capitis Posterior Major and the Obliquus Capitis Inferior resulted in C2 nerve tension due to the myodural bridge.

This cadaver study cannot be used to determine the clinical consequences of the collagenous connections between the nuchal ligament and the dural sac and the myodural bridges (with and without movement). However, these connections might be able to help explain cervical-cephalic pain and conditions such as cervicogenic and tension type headaches. Cervical pain patients can present with a variety of musculoskeletal myofascial syndromes in the upper quadrant and reduced active and passive movements (Borghouts, Koes & Bouter, 1998). The presence of the myodural bridges between the suboccipital muscles and the dura might play a role and contribute to the pathogenesis and therefore needs to be further evaluated (Scali et al., 2015; Enix, Scali & Pontell, 2014).

A possible drawback of this study was the use of formalin-embalmed specimens were used. It is well-known that the fixation and desiccation effects of formalin solution can cause tissue dehydration and shrinkage and might have affected the presentation and density of the tissues we observed (Chen et al., 2015). This might have affected the effect of the forceps pull on the suboccipital muscles and nuchal ligament on the dura. Only seven cadavers were used in this study because the Department of Rehabilitation Sciences at Florida Gulf Coast University only receives 7 cadavers annually for educational purposes. This limited number of cadavers does limit the generalizability of our results. Additionally, the cadavers received from the Anatomical Board of the State of Florida were all over the age of 50 and medical history was not known. Normal aging can have an effect on anatomical structures, and this could have influenced our findings.

## Conclusion

Our cadaver study confirmed the presence of a large network of tissue connections between ligament, bone and muscles in the suboccipital region. There was a fairly wide myodural bridge between the Rectus Capitis Major, Minor and the Obliquus Capitis Inferior and dura sac in the spinal canal. We observed that the nuchal ligament was continuous with the meningovertebral ligament between C2 and the dural sac. Histology revealed this connection was high in collagenous tissue similar to that of the nuchal ligament and the dura. Additionally, a clear connective tissue strand with the same tissue formation was identified between the posterior arch of C1 and the dura. It was demonstrated that passive motion of the upper cervical spine and C1 results in a change of position of the spinal cord within the spinal canal and resulting in tightening of the myodural, meningovertebral ligaments, and spinal nerves. Future studies should further evaluate this relationship and determine the clinical significance.

## Supplemental Information

10.7717/peerj.9716/supp-1 Supplemental Information 1 Observational table identifies which cadavers had the reported findings.

## Additional Information and Declarations

### Data Availability

The following information was supplied regarding data availability: This was a dissection observational study. Observations are available in a Supplemental File.
