# Supplementary material for: Anatomy and clinical relevance of sub occipital soft tissue connections with the dura mater in the upper cervical spine
Source: PeerJ. 2020 Aug 10;8:e9716. doi: 10.7717/peerj.9716 (PMC7425638; doi:10.7717/peerj.9716)
Supplement: Supplemental Information 1 [file peerj-08-9716-s001.docx]

| cadaver | Myo  RCP Ma | Myo  RCP Mi | Myo  OCi | Nuchal spine | Menigio-vert fuzz |
| --- | --- | --- | --- | --- | --- |
| 1 | + | + | + | + | + |
| 2 | + | + | + | + | + |
| 3 | + | + | + | + | + |
| 4 | + | + | + | + | + |
| 5 | + | + | + | + | + |
| 6 | + | + | + | + | + |
| 7 | + | + | + | + | + |

| cadaver | flexion | extension | Rot right | Rot left |
| --- | --- | --- | --- | --- |
| 1 | backward | forward | right | left |
| 2 | backward | forward | right | left |
| 3 | backward | forward | right | left |
